# Support Effects of Microwave-Synthesized Ru-Based Catalysts on Their Hydrogen Evolution Performance in Acidic Media

**DOI:** 10.3390/nano16020097

**Published:** 2026-01-12

**Authors:** Luan Liu, Hongru Liu, Genghua Cao, Xiaoyu Wu, Baorui Jia, Lin Su, Linhui Su, Xuanhui Qu, Mingli Qin

**Affiliations:** 1 Institute for Advanced Materials and Technology, University of Science and Technology Beijing, Beijing 100083, China; b2230886@ustb.edu.cn (L.L.); qfliuhongru@163.com (H.L.); quxh@ustb.edu.cn (X.Q.); 2 School of Automotive and Transportation Engineering, Shenzhen Polytechnic University, Shenzhen 518055, China; caogenghua@szpu.edu.cn (G.C.);; 3 Department of Materials Science and Engineering, National University of Singapore, Singapore 117575, Singapore; 4 Shunde Innovation School, University of Science and Technology Beijing, Foshan 301811, China; 5 Yunnan Hongsheng Technology of Platinum-New Materials Co., Ltd., Yuxi 653100, China; linhuisu2020@163.com; 6 Faculty of Material Science and Engineering, Kunming University of Science and Technology, Kunming 650093, China; 7 Beijing Advanced Innovation Center for Materials Genome Engineering, University of Science and Technology Beijing, Beijing 100083, China; 8 Institute of Materials Intelligent Technology, Liaoning Academy of Materials, Shenyang 110167, China

**Keywords:** Ru-based catalysts, TiO_2_ support, hydrogen evolution reaction (HER), acidic media, metal–support interactions

## Abstract

Ruthenium-based catalysts supported on TiO_2_, SnO_2_, and WO_3_ were synthesized via a microwave-assisted rapid reduction method and evaluated for the hydrogen evolution reaction (HER) in acidic media. The Ru species existed as highly dispersed nanoclusters, as confirmed by XRD and TEM, and the catalytic activity was strongly dependent on the oxide support. Ru/TiO_2_ exhibited the best HER performance, achieving an overpotential of 187 mV at 10 mA·cm^−2^ and a Tafel slope of 97.56 mV·dec^−1^. While particle size differences (1.8–3.7 nm) did not account for the activity trend, XPS revealed distinct metal–support interactions that modulated the electronic state of Ru. Ru/TiO_2_ showed an intermediate electron depletion that optimizes the Ru-H binding strength, explaining its superior kinetics. Regulation of Ru loading further identified Ru/15TiO_2_ as the optimal catalyst, exhibiting low charge transfer resistance and excellent stability over 17 h. This study highlights the critical role of support-induced electronic modulation and loading engineering in designing efficient Ru-based electrocatalysts for acidic HER.

## 1. Introduction

Hydrogen energy, as a clean secondary energy source with high energy density, is considered one of the most promising pathways to achieve carbon neutrality [1,2,3]. Among the various hydrogen production technologies, water electrolysis has attracted extensive attention due to its advantages of high hydrogen purity and environmental friendliness [4,5,6,7]. However, the hydrogen evolution reaction (HER) involved in water splitting suffers from sluggish kinetics, necessitating highly efficient and stable electrocatalysts to reduce energy consumption and enhance efficiency [8,9,10]. Currently, Pt-based materials are regarded as the most effective HER catalysts, but their widespread application is severely hindered by their high cost and limited natural abundance. Consequently, the development of non-Pt catalysts with both cost-effectiveness and outstanding performance has become a research hotspot [11,12,13,14].

Among the noble metals, ruthenium (Ru) is regarded as one of the most promising alternatives because of its relatively abundant reserves, much lower price compared to Pt, and nearly optimal hydrogen adsorption free energy [15,16,17]. Nevertheless, Ru catalysts still face two critical challenges: limited stability under acidic conditions and the tendency of Ru nanoparticles to agglomerate at high loadings, which leads to reduced catalytic activity [18]. Therefore, it is of great scientific importance to develop rational material design strategies to improve the utilization efficiency and stability of Ru-based catalysts [19,20].

The support effect has been demonstrated as an effective strategy to enhance the performance of metal catalysts [21,22]. Different oxide supports can regulate the electronic structure [23,24,25] of active sites and catalytic processes through electronic effects, interfacial interactions [26], and surface structural modifications [27]. Previous studies have reported that common oxides such as TiO_2_ [28,29,30], SnO_2_ [31,32], and WO_3_ [33,34] not only help disperse metal nanoparticles but also improve charge transfer characteristics via metal–support interactions [35,36], thereby optimizing electrocatalytic performance. However, the performance variation and underlying mechanism of Ru-based catalysts [21,22] on different supports remain insufficiently understood, especially in the context of HER under acidic conditions.

In addition, optimizing the Ru loading is equally crucial. Insufficient loading leads to a lack of active sites, while excessive loading often results in nanoparticle agglomeration or blockage of the support surface [37], thereby reducing the utilization efficiency of active sites. Thus, exploring the optimal Ru loading in different support systems is of great significance for achieving efficient and durable HER performance.

Microwave-assisted synthesis has been widely applied for the rapid fabrication of supported noble-metal catalysts, enabling ultrafast nucleation, uniform energy deposition, and effective suppression of particle growth, which collectively favor high metal dispersion even at relatively high loadings [38,39,40,41]. Microwave-assisted synthesis has been reported to differ from conventional thermal treatment mainly in the mode of heat delivery. Conventional heating relies on relatively slow external heat conduction, which readily leads to temperature gradients and is typically accompanied by prolonged nucleation-growth processes [42,43]; in contrast, microwave irradiation enables rapid and homogeneous volumetric heating throughout the entire reaction system. It has been widely recognized that these differences in heating characteristics are generally considered to influence nucleation and growth processes during catalyst formation, with potential implications for metal dispersion and metal–support interactions [42,44,45].

Despite these advances, systematic investigations into support-dependent electronic modulation and Ru loading effects under acidic HER conditions remain limited. In this work, microwave-assisted synthesis is employed to ensure identical preparation conditions, enabling a controlled comparative study of Ru-based catalysts.

Motivated by these considerations, in this study, Ru-based catalysts supported on TiO_2_, SnO_2_, and WO_3_ were successfully synthesized using a microwave-assisted rapid reduction method [38,39,40,41,46].

Remarkably, the entire synthesis process can be completed within one minute, offering advantages of simplicity, low energy consumption, and suitability for large-scale preparation. Through systematic structural and electrochemical characterizations, we revealed the significant support effect on the HER performance of Ru-based catalysts and identified TiO_2_ as the most efficient support. Furthermore, optimization of Ru loading on TiO_2_ demonstrated that an appropriate loading amount achieves the best balance between catalytic activity and stability. This work not only provides new insights into the support effect of Ru-based catalysts but also offers a feasible strategy for designing highly efficient and cost-effective HER electrocatalysts in acidic media.

## 2. Experimental Section

### 2.1. Materials

Ruthenium(III) chloride (RuCl_3_) and tin(IV) oxide (SnO_2_) were purchased from Shanghai Macklin Biochemical Co., Ltd., Shanghai, China. Tungsten trioxide (WO_3_) and titanium dioxide (TiO_2_) were obtained from Hebei Yuanying New Material Co., Ltd., Hebei, Xingtai, China. Sodium dihydrogen phosphate dihydrate (NaH_2_PO_4_·2H_2_O) was used as the reducing agent, which was purchased from Wuxi Zhanwang Chemical Co., Ltd., Wuxi, China. All chemicals were of analytical grade and used without further purification.

### 2.2. Synthesis of Ru/MO_x_ Catalysts

Ru-based catalysts supported on different oxides (Ru/MO_x_, MO_x_ = WO_3_, SnO_2_, TiO_2_) were synthesized via a rapid microwave-assisted reduction method. In a typical procedure, 15 mg of MO_x_ powder, 10.6 mg of RuCl_3_, and 10.6 mg of NaH_2_PO_4_·2H_2_O were thoroughly ground in a mortar to achieve a homogeneous mixture. The mixture was then transferred into a 5 mL quartz vial, wetted with deionized water, and subjected to microwave irradiation at 700 W for 1 min. The obtained product was collected, ground into powder, and subsequently calcined in air at 400 °C for 0.5 h.

For Ru/TiO_2_ catalysts with different Ru loadings, the same procedure was followed except for the TiO_2_ content. Specifically, 5 mg, 15 mg, 30 mg, and 60 mg of TiO_2_ were used to prepare Ru/5TiO_2_, Ru/15TiO_2_, Ru/30TiO_2_, and Ru/60TiO_2_, respectively. While keeping the Ru precursor (RuCl_3_, 10.6 mg) and reducing agent (NaH_2_PO_4_·2H_2_O, 10.6 mg) constant. Therefore, these samples were designed to systematically adjust the Ru-to-support ratio in order to optimize the dispersion and interfacial interaction of Ru species on TiO_2_.

### 2.3. Characterization

The phase composition of the products was identified by X-ray diffraction (XRD, Rigaku D/max-RB12, Rigaku, Tokyo, Japan) using Cu Kα radiation in the 2θ range of 10–80° at room temperature. The morphology of the samples was examined by field-emission scanning electron microscopy (FESEM, NOVA NANOSEM 450, FEI, Hillsboro, OR, USA). TEM, HRTEM images, HAADF-STEM, and EDS mapping data were obtained using a JEOL JEM-2010 transmission electron microscope (JEOL, Tokyo, Japan) operated at an accelerating voltage of 300 kV. The inductively coupled plasma mass spectrometry (ICP-MS) was performed on an Agilent cOES730 system (Agilent, Santa Clara, CA, USA) using argon as the carrier gas.

### 2.4. Electrochemical Performance Characterization

Electrochemical measurements were carried out in 0.5 M H_2_SO_4_ solution using a conventional three-electrode system. A saturated calomel electrode (SCE), a graphite rod, and a glassy carbon electrode (GCE, 3 mm in diameter) were employed as the reference electrode, counter electrode, and working electrode, respectively. To prepare the working electrode, 5 mg of catalyst and 20 μL of 5 wt% Nafion solution were dispersed in a mixed solvent of 480 μL deionized water and 480 μL ethanol, followed by ultrasonication for 0.5 h to obtain a homogeneous catalyst ink. Then, 4 μL of the ink was drop-cast onto the surface of the GCE to serve as the working electrode. (For chronoamperometric stability tests, 50 μL of the catalyst ink was uniformly coated onto a 1 × 1 cm^2^ piece of carbon paper, which was used as the working electrode.) All electrochemical tests were conducted on a CHI 660E electrochemical workstation (CH Instruments, Inc., Shanghai, China). LSV data were collected at a scan rate of 5 mV s^−1^. All potentials were calibrated to the reversible hydrogen electrode (RHE) using the equation:

E_RHE_ = E_SCE_ + 0.242 V + 0.059 × pH

The current density (j) was normalized to the geometric area of the GCE and used to construct Tafel plots. Electrochemical impedance spectroscopy (EIS) measurements of the catalysts under HER conditions were performed at an initial potential of −0.41 V for direct comparison. The AC perturbation amplitude was 5 mV, and the frequency range was swept from 100 kHz to 1 Hz. The EIS responses of all electrodes were fitted using a simplified Randles equivalent circuit. The electrochemically active surface area (ECSA) was determined based on electrochemical double-layer capacitance (C_dl_) to assess the number of active sites of a HER electrocatalyst. In the cyclic voltammetry (CV) experiments, the potential window ranged from 0.85 to 0.95 V vs. RHE, with scan rates varying from 20 to 120 mV s^−1^. The C_dl_ was determined using the following equation:

$$∆j_{c}\times A=vC_{dl}$$

where v represents the scan rate and Δj_c_ denotes the difference in current density at 0.78 V vs. RHE, and A stands for the surface area of GCE (0.07065 cm^−2^). The slope of the plot of Δj_c_ × A versus v is twice the value of C_dl_.

## 3. Results and Discussion

**Figure 1:**
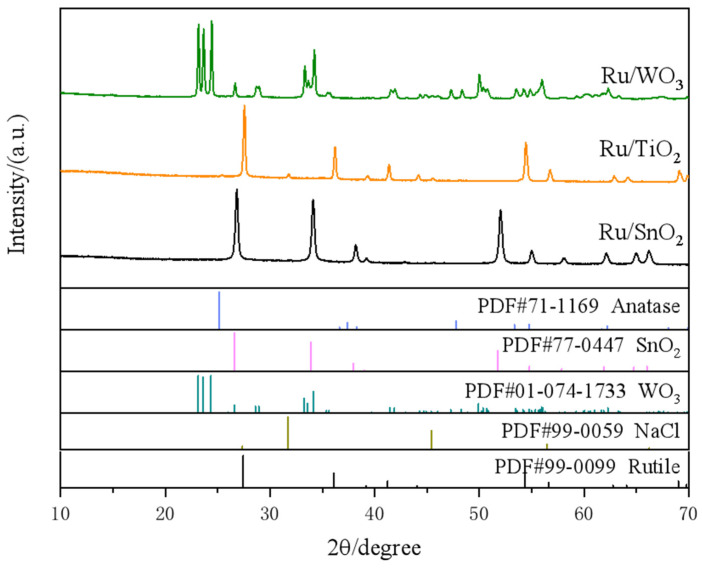
XRD patterns of Ru catalysts supported on different oxide supports, compared with the corresponding standard reference diffraction patterns (PDF cards) of the oxide supports.

The Ru-based catalysts investigated in this work were synthesized via a rapid microwave-assisted reduction strategy, in which the entire reaction is completed within one minute. This method provides uniform and rapid energy input, favoring fast nucleation and effective dispersion of Ru species on oxide supports, which is critical for interpreting the following structural and electrochemical analyses. To gain insight into the structure of the Ru-based supported catalysts, XRD characterization was performed. All peaks are indexed to the corresponding support phases (Figure 1). A peak near ~44° in Ru/WO_3_ or Ru/TiO_2_ may include contributions from Ru(101) but overlap, and low Ru content prevents an unambiguous assignment, which is mainly attributed to the ultra-small size of Ru species and their high dispersion on the support. The other diffraction peaks observed in Figure 1 are assigned to the crystalline supports themselves. The NaCl reference diffraction pattern is included solely for phase-identification completeness. Although extremely weak diffraction features can be observed at angles corresponding to NaCl, their intensities are close to the background noise level and are insufficient to indicate the presence of a distinct crystalline NaCl phase. These trace signals are attributed to residual sodium-containing species formed during the synthesis process and have a negligible influence on structural identification and catalytic performance. Following the XRD analysis, the actual Ru loadings of the catalysts were quantified by ICP-MS. The measured Ru contents were 21.36 wt% for Ru/SnO_2_, 19.50 wt% for Ru/TiO_2_, and 21.16 wt% for Ru/WO_3_ (Table S1), all of which are close to the theoretical value (21.46 wt%) calculated from the precursor stoichiometry, indicating accurate control of the Ru loading during synthesis and enabling a reliable comparison of support effects in subsequent electrochemical analyses.

**Figure 2:**
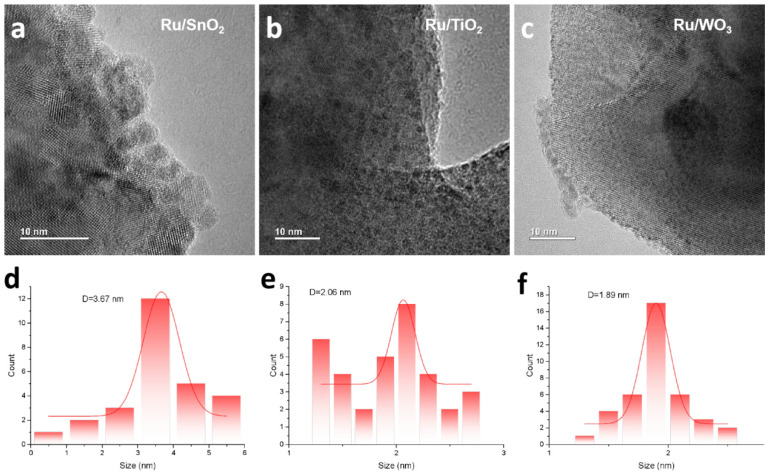
(**a**–**c**) TEM images of Ru catalysts supported on different oxides; (**d**–**f**) the corresponding particle size distributions of Ru nanoparticles on the respective supports shown in (**a**–**c**).

The particle size distributions of Ru catalysts supported on different oxides were determined by transmission electron microscopy (TEM). As shown in Figure 2, the average particle sizes of Ru species varied among the different catalysts. All Ru nanoparticles are uniformly distributed on the oxide supports without noticeable aggregation, indicating efficient dispersion achieved by the microwave-assisted reduction. Since all samples were synthesized using the same method and the same Ru precursor, the differences in Ru particle sizes are expected to originate from the structural or electronic modulation of Ru active centers induced by different supports. The average Ru particle sizes decreased in the following order: Ru/SnO_2_ (3.67 nm) > Ru/TiO_2_ (2.06 nm) > Ru/WO_3_ (1.89 nm). According to the TEM results, Ru exists in the form of highly dispersed nanoclusters, whose XRD diffraction signals are too weak to be distinguished from the strong diffraction peaks of the catalyst supports.

**Figure 3:**
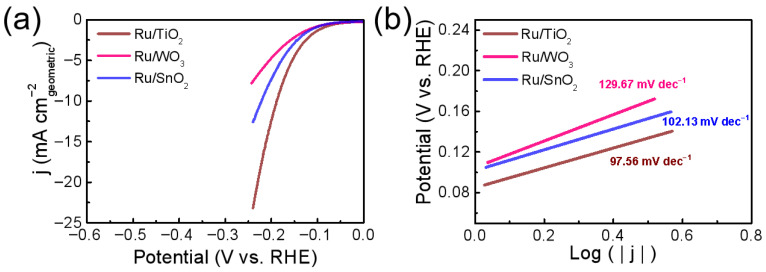
(**a**) Linear sweep voltammetry (LSV) curves (without iR correction) of Ru-based catalysts supported on different oxides in 0.5 M H_2_SO_4_ solution; (**b**) the corresponding Tafel plots of Ru-based catalysts supported on different oxides.

To further evaluate the electrocatalytic activity of Ru supported on different oxide catalysts toward the hydrogen evolution reaction (HER) at room temperature, electrochemical measurements were carried out in a typical three-electrode system, with a graphite rod as the counter electrode. Linear sweep voltammetry (LSV) tests were conducted in 0.5 M H_2_SO_4_, and the corresponding polarization curves without iR correction are shown in Figure 3a. Among the catalysts, Ru/WO_3_ exhibited the lowest HER activity, while Ru/SnO_2_ showed moderate catalytic performance. In contrast, Ru/TiO_2_ required an overpotential of only 187 mV (η) to achieve a current density of 10 mA·cm^−2^, which is significantly lower than that of Ru/SnO_2_ (219 mV), indicating a stronger interaction between Ru and the TiO_2_ support. Based on the LSV results, the strength of Ru-support interactions follows the order: TiO_2_ > SnO_2_ > WO_3_. Since all catalysts were prepared with the same nominal Ru content, the performance difference mainly arises from the support effect rather than Ru loading, such as the support-induced modulation of the electronic structure and interfacial properties. The similar Ru loadings (Table S1) indicate that the HER activity differences mainly arise from support effects rather than Ru loading.

The HER kinetics were further investigated using the corresponding Tafel plots (η vs. log j), fitted by the equation η = a + b log (j), where b is the Tafel slope, and a is the intercept. As presented in Figure 3b, the Ru/TiO_2_ catalyst exhibited the lowest Tafel slope (97.56 mV·dec^−1^), whereas Ru/SnO_2_ (102.13 mV·dec^−1^) and Ru/WO_3_ (129.67 mV·dec^−1^) showed higher values, indicating more sluggish kinetics. Overall, the results reveal that the support exerts a significant influence on the HER performance of Ru catalysts, with TiO_2_-supported Ru demonstrating more favorable reaction kinetics and superior catalytic activity. A lower Tafel slope generally corresponds to a smaller reaction overpotential and a higher rate constant, reflecting more efficient hydrogen adsorption–desorption behavior on the catalyst surface. To further understand the effect of the support and Ru loading on the electrochemically active surface area (ECSA), cyclic voltammetry (CV) measurements were performed in the non-faradaic potential region, and the double-layer capacitance (C_dl_) values were extracted to estimate the ECSA (Figure S1). The C_dl_ values follow the trend Ru/TiO_2_ > Ru/WO_3_> Ru/SnO_2_, indicating that Ru/TiO_2_ possesses the largest electrochemically active surface area and the greatest number of accessible catalytic sites. Although Ru/TiO_2_ exhibits a larger ECSA, its superior HER performance is also reflected in lower Tafel slopes and charge transfer resistance, indicating enhanced intrinsic activity beyond surface area effects. This suggests that the TiO_2_ support provides a more favorable surface structure and interfacial wettability, facilitating more effective exposure of Ru active centers to the electrolyte. Overall, the results reveal that the support exerts a significant influence on the HER performance of Ru catalysts, with TiO_2_-supported Ru demonstrating more favorable reaction kinetics and superior catalytic activity.

**Figure 4:**
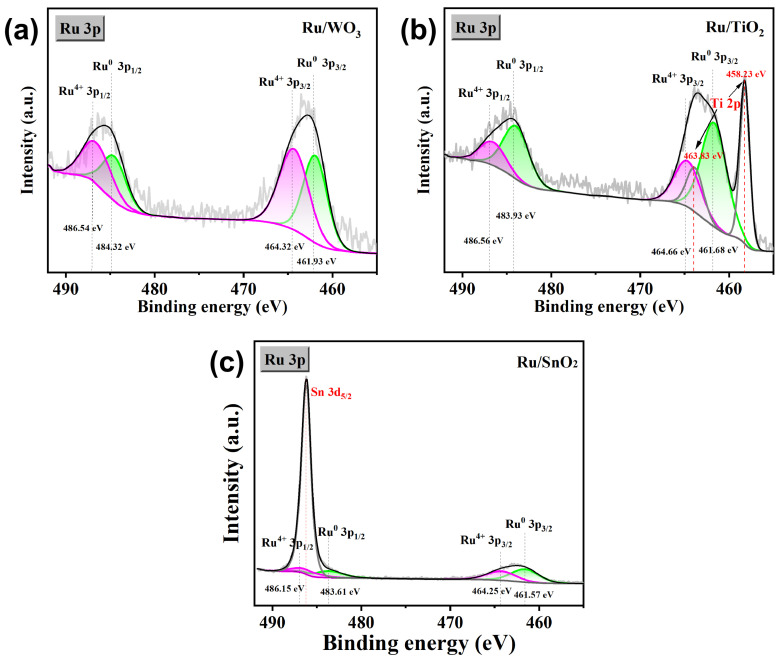
High-resolution Ru 3p XPS spectra of (**a**) Ru/WO_3_, (**b**) Ru/TiO_2_ and (**c**) Ru/SnO_2_ catalysts.

To further elucidate the support-dependent electronic effects, high-resolution Ru 3p_3_/_2_ spectra were analyzed for the three catalysts (Figure 4). The binding energy of metallic Ru^0^ follows the order Ru/SnO_2_ (461.57 eV) < Ru/TiO_2_ (461.68 eV) < Ru/WO_3_ (461.93 eV), indicating that Ru is most electron-rich on SnO_2_ and most electron-deficient on WO_3_. Importantly, as shown in Table 1, Ru/TiO_2_ exhibits an intermediate Ru^0^ binding energy together with the highest proportion of metallic Ru^0^ (69.4%), suggesting a moderately electron-depleted state with abundant active metal sites. Such an optimized electronic configuration effectively tunes the intrinsically strong Ru-H interaction toward a more thermoneutral ΔG_H*_, thereby facilitating faster HER kinetics. This electronic trend is fully consistent with the HER activity sequence (Ru/TiO_2_ > Ru/SnO_2_ > Ru/WO_3_), confirming that TiO_2_ induces the most favorable metal–support interaction for enhancing Ru-based catalytic performance. The detailed Ru 3p peak-fitting parameters, including binding energies, FWHM values, and peak areas for Ru^0^ and Ru^4+^ species, are summarized in Table S2 in the Supporting Information. The O 1 s spectra were deconvoluted into lattice oxygen (O_at_), defect-related oxygen species (O_def_), and surface-adsorbed oxygen (O_ad_), as shown in Figure S3 and Table S3. The relative contribution of O_def_ follows the order Ru/TiO_2_ (53.76%) > Ru/WO_3_ (47.58%) > Ru/SnO_2_ (45.45%), indicating distinct support-dependent oxygen chemical environments. Together with the Ru 3p results, these observations suggest that the oxide supports participate in metal–support interactions and contribute to the electronic modulation of Ru. High-resolution XPS spectra of the support elements (Ti 2p, Sn 3d, and W 4f) were collected to examine the chemical states of the oxide substrates after Ru deposition (Figure S4 and Table S4). The Ti 2p peaks at 458.49 and 464.19 eV correspond to Ti^4+^ in TiO_2_, the Sn 3d_5_/_2_ and Sn 3d_3_/_2_ peaks at 486.82 and 495.82 eV are characteristic of Sn^4+^ in SnO_2_, and the W 4f_7_/_2_ and W 4f_5_/_2_ peaks at 36.01 and 38.14 eV are assigned to W^6+^ in WO_3_. No distinct low-valence species were detected, indicating that the oxide supports largely preserve their stable oxidation states. Together with the Ru 3p results, these observations further indicate that the oxide supports provide distinct interfacial environments for metal–support interactions.

Although the Ru nanoparticles exhibited obvious size differences on different supports, the catalytic performance did not follow a clear trend with particle size. This indicates that the catalytic activity is not solely dependent on particle size but is also influenced by multiple factors, including the electronic structure of the supports, metal–support interactions, and the accessibility of surface active sites. Therefore, particle size is not the only dominant factor determining the catalytic performance.

**Figure 5:**
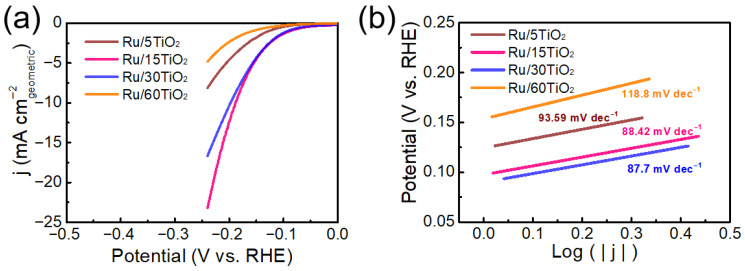
(**a**) Linear sweep voltammetry (LSV) curves of Ru/TiO_2_ catalysts with different TiO_2_ loadings (5, 15, 30, and 60 mg) in acidic electrolyte; (**b**) the corresponding Tafel plots.

Notably, the Ru/TiO_2_ catalyst discussed in the support-effect section corresponds to the Ru/15TiO_2_ sample in the Ru-loading-dependent study, as both were synthesized using 15 mg of TiO_2_ with an identical Ru precursor amount. These two sections therefore represent complementary analyses of the same material from different perspectives. Since the Ru/TiO_2_ catalyst exhibited superior catalytic activity toward the hydrogen evolution reaction (HER), a systematic study was conducted to investigate the effect of Ru loading on its performance. The actual Ru contents of the Ru/TiO_2_ catalysts were further verified by ICP-MS, and the measured values closely matched the theoretical loadings (Table S5). Figure 5a shows the linear sweep voltammetry (LSV) curves of Ru/TiO_2_ catalysts with different Ru loadings. It can be observed that the current density increased with increasing Ru loading, indicating enhanced HER activity. Among them, Ru/15TiO_2_ displayed a significantly higher current density at the same overpotential compared with Ru/30TiO_2_, Ru/5TiO_2_ and Ru/60TiO_2_, suggesting that a moderate increase in Ru loading can markedly improve catalytic activity. However, when the loading was excessively high (Ru/60TiO_2_), the HER performance decreased, which may be attributed to Ru nanoparticle agglomeration or the reduced utilization efficiency of active sites. Thus, an appropriate Ru loading is beneficial for optimizing catalytic performance.

Figure 5b presents the Tafel plots of Ru/TiO_2_ catalysts with different Ru loadings. The Tafel slopes varied with loading: 93.59, 88.42, 87.70, and 118.80 mV·dec^−1^ for Ru/5TiO_2_, Ru/15TiO_2_, Ru/30TiO_2_, and Ru/60TiO_2_, respectively. It is evident that catalysts with moderate Ru loadings (15–30 wt%) exhibited lower Tafel slopes, indicating faster electrochemical kinetics. In contrast, either too low or too high Ru loading resulted in higher Tafel slopes, implying that excessive Ru loading may decrease the utilization efficiency of active sites, thereby weakening the overall electrocatalytic performance. Furthermore, for Ru/TiO_2_ catalysts with different Ru loadings, the C_dl_ values decrease in the order Ru/15TiO_2_ > Ru/30TiO_2_ > Ru/5TiO_2_ > Ru/60TiO_2_, demonstrating that a moderate Ru loading (15 mg TiO_2_) yields the highest accessible electrochemical surface area (Figure S2). These results indicate that both the nature of the support and the Ru loading critically influence the number of active sites and, consequently, the overall HER performance. 

**Figure 6:**
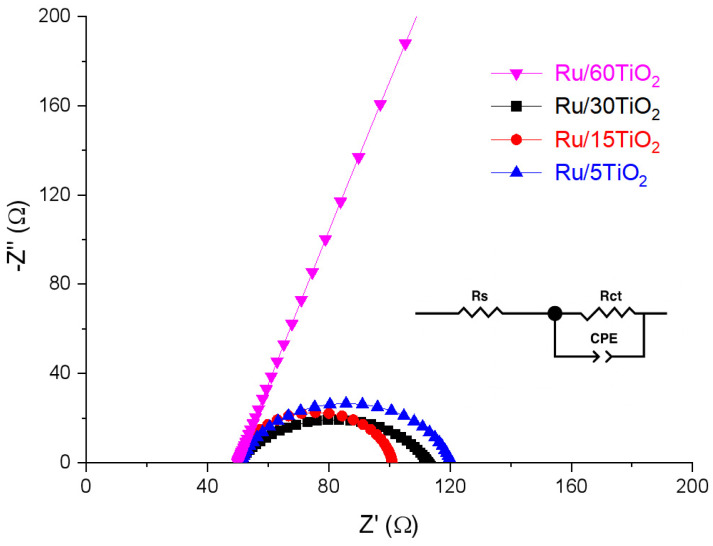
Nyquist plots of different working electrodes at −410 mV in 0.5 M H_2_SO_4_; the frequency was swept from 100 kHz to 1 Hz (from high to low frequency); the inset shows the equivalent circuit model used for fitting the impedance spectra.

**Figure 7:**
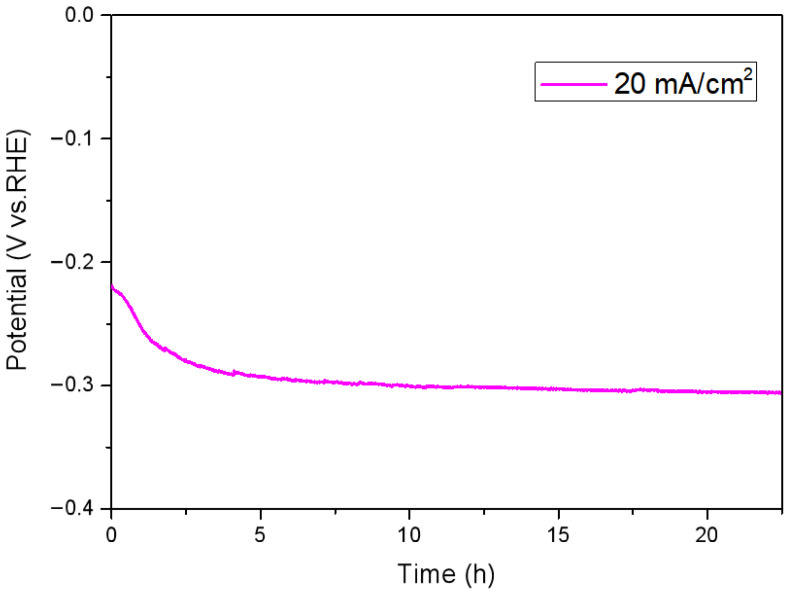
Potential–time (V-t) stability curve of Ru/15TiO_2_ at a current density of 20 mA cm^−2^ in 0.5 M H_2_SO_4_.

Next, electrochemical impedance spectroscopy (EIS) was employed to investigate the charge transfer between the catalysts and the electrolyte. The Nyquist plots of Ru/TiO_2_ catalysts with different Ru loadings are shown in Figure 6. In these spectra, the ohmic resistance (Rs) extracted from the high-frequency intercepts was around 50 Ω for all electrodes, indicating that Rs is mainly governed by the electrolyte and cell geometry, and its small variation does not significantly affect the comparison of HER activities among different catalysts. And the diameter of the semicircle in the low-frequency region represents the charge transfer resistance (Rct) at the catalyst-electrolyte interface. It can be observed that the samples with different Ru loadings exhibited distinct electrochemical impedance characteristics. The charge transfer resistance of Ru/15TiO_2_ was approximately 50 Ω, while the Rct of the Ru/30TiO_2_ was about 62 Ω, with both being lower than that of Ru/5TiO_2_ (70 Ω). In contrast, Ru/60TiO_2_ exhibited the largest charge transfer resistance among the four samples, suggesting sluggish interfacial electron transfer. The sharply reduced interfacial charge transfer barrier is consistent with the high HER activity of Ru/15TiO_2_. To further evaluate the stability of Ru/15TiO_2_, chronoamperometric measurements were performed at a constant current density of 20 mA·cm^−2^ in 0.5 M H_2_SO_4_ (Figure 7). The potential exhibited a slight initial increase within the first 2 h, which can be attributed to the activation and surface restructuring of Ru species under reductive conditions. After this short activation stage, the potential gradually stabilized and remained nearly constant (around −0.30 V vs. RHE) for over 17 h, indicating excellent durability and strong metal–support interactions between Ru nanoparticles and TiO_2_. The negligible potential drift during the extended test demonstrates that the Ru active sites are highly stable against dissolution or agglomeration in the acidic medium, confirming the robustness of the catalyst under continuous HER operation.

**Figure 8:**
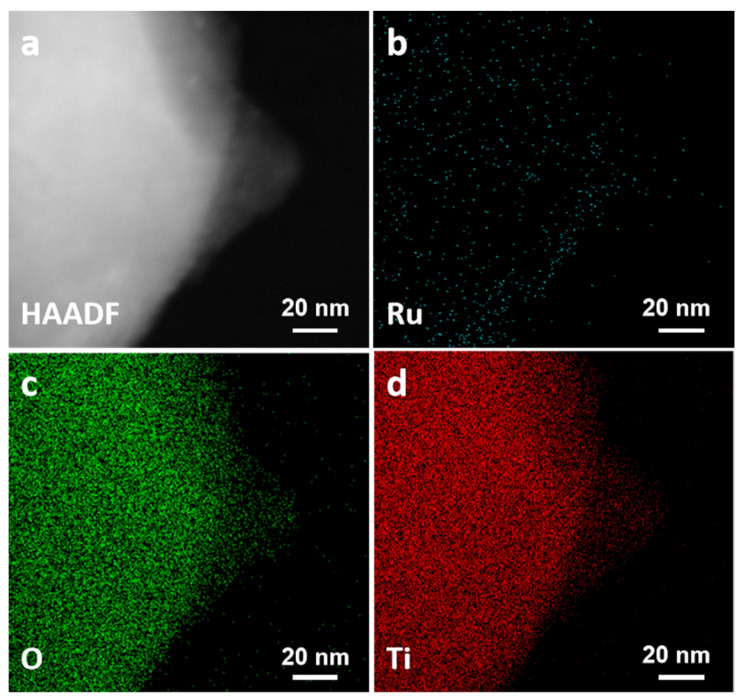
(**a**) HAADF-STEM image of Ru/15TiO_2_; (**b**–**d**) the corresponding elemental mapping images of Ru, O, and Ti, respectively.

**Figure 9:**
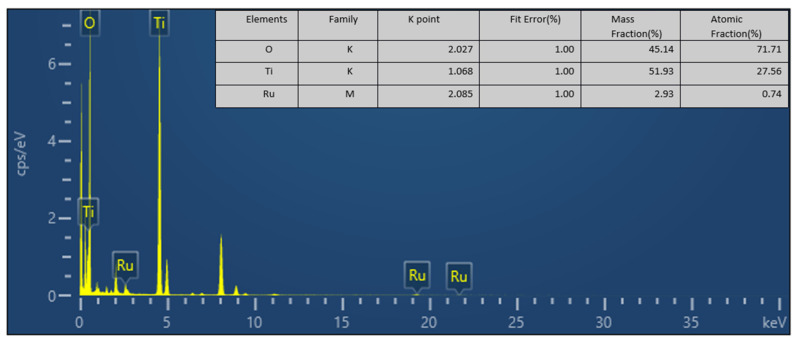
EDS spectrum and elemental quantitative analysis of Ru/15TiO_2_.

The elemental composition of the particles was investigated using HAADF-STEM-EDS elemental mapping analysis. As shown in Figure 8, HAADF-STEM-EDS suggests no large Ru-rich agglomerates on TiO_2_. And the weak, diffuse Ru signal is consistent with the low Ru content (Figure 9), EDS quantitative analysis revealed that the mass ratio of Ti to Ru was approximately 18:1, with a molar ratio of about 37:1, confirming the low Ru loading (Figure 9).

## 4. Conclusions

This work demonstrates that the catalytic behavior of Ru toward acidic HER is governed predominantly by support-induced electronic modulation rather than particle size or dispersion alone. Through a one-minute microwave-assisted synthesis, Ru nanoclusters were anchored onto TiO_2_, SnO_2_, and WO_3_ supports, revealing TiO_2_ as the most effective platform. The superior performance of Ru/TiO_2_ originates from a support-engineered, moderately electron-depleted Ru state that optimizes the Ru–H binding strength and accelerates interfacial charge transfer. Loading-dependent studies further reveal that Ru/15TiO_2_ provides the optimal configuration of active-site accessibility, electronic structure, and stability, sustaining long-term operation at 20 mA·cm^−2^. Collectively, this work establishes a clear design principle: precise control of metal–support electronic interactions, coupled with rational tuning of active-metal loading, is essential for maximizing the intrinsic reactivity of Ru-based HER catalysts. These insights offer a generalizable strategy for developing next-generation, low-PGM electrocatalysts through support engineering and rapid microwave synthesis.

## Figures and Tables

**Table 1:** Summary of Ru^0^ 3p_3_/_2_ binding energy and metallic Ru fraction for Ru/WO_3_, Ru/TiO_2_ and Ru/SnO_2_ catalysts.

Catalyst	Ru^0^ 3p_3_/_2_ BE (eV)	Metallic Ru^0^ (%)
Ru/WO_3_	461.93	46.5
Ru/TiO_2_	461.68	69.37
Ru/SnO_2_	461.57	57.61

## Data Availability

The original contributions presented in this study are included in the article/Supplementary Materials. Further inquiries can be directed to the corresponding authors.

## Supplementary Materials

The following supporting information can be downloaded at: https://www.mdpi.com/article/10.3390/nano16020097/s1, Figure S1. CV curves measured at different scan rates from 20 to 120 mV s^−1^ in 0.5 M H_2_SO_4_ for (a) Ru/WO_3_, (b) Ru/TiO_2_, (c) Ru/SnO_2_ (d) Capacitive current at middle potential of CV curves as function of scan rates for Ru/WO_3_, Ru/TiO_2_ and Ru/SnO_2_. Figure S2. (a–d)CV curves measured at different scan rates from 20 to 120 mV s^−1^ in 0.5 M H_2_SO_4_ for different catalysts. (e) Capacitive current at middle potential of CV curves as function of scan rates for different catalysts. Figure S3. High-resolution O_1s_ XPS spectra of (a) Ru/WO_3_, (b) Ru/TiO_2_ and (c) Ru/SnO_2_ catalysts. Figure S4. High-resolution XPS spectra of the support elements for Ru-supported oxide catalysts: (a) W 4f spectrum of Ru/WO_3_, (b) Ti 2p spectrum of Ru/TiO_2_, and (c) Sn 3d spectrum of Ru/SnO_2._ Table S1. ICP-MS analysis of the synthesized catalysts together with theoretical mass loadings. The theoretical values correspond to the wt% used in the synthesis. Table S2. Detailed Ru 3p peak-fitting parameters for the three catalysts. Table S3. Detailed O 1s peak-fitting parameters for Ru/MO_x_ catalysts. Table S4. Binding energies of support element core levels for Ru-supported oxide catalysts. Table S5. ICP-MS analysis of the synthesized catalysts together with theoretical mass loadings. The theoretical values correspond to the wt% used in the synthesis.
