# Supplementary material for: Support Effects of Microwave-Synthesized Ru-Based Catalysts on Their Hydrogen Evolution Performance in Acidic Media
Source: Nanomaterials (Basel). 2026 Jan 12;16(2):97. doi: 10.3390/nano16020097 (PMC12844415; doi:10.3390/nano16020097)
Supplement: Supplementary file 1 [file nanomaterials-16-00097-s001.zip › nanomaterials-4061628-supplementary.pdf]

Supplementary Information

Support Effects of Microwave-Synthesized Ru-Based Catalysts on Their Hydrogen Evolution Performance in Acidic Media

Luan Liu [1,†], Hongru Liu [1,†], Genghua Cao [2], Xiaoyu Wu [2], Baorui Jia [1,3,4,*], Lin Su [5,6,*], Linhui Su [5,6], Xuanhui Qu [1,7] and Mingli Qin [1,7,8,*]

[1] Institute for Advanced Materials and Technology, University of Science and Technology Beijing, Beijing 100083, China; b2230886@ustb.edu.cn (L.L.); qfliuhongru@163.com (H.L.); quxh@ustb.edu.cn (X.Q.)

[2] School of Automotive and Transportation Engineering, Shenzhen Polytechnic University, Shenzhen 518055, China; caogenghua@szpu.edu.cn (G.C.); wuxiaoyu@szpu.edu.cn (X.W.)

[3] Department of Materials Science and Engineering, National University of Singapore, Singapore 117575, Singapore

[4] Shunde Innovation School, University of Science and Technology Beijing, Foshan 301811, China

[5] Yunnan Hongsheng Technology of Platinum-New Materials Co., Ltd., Yuxi 653100, China; linhuisu2020@163.com

[6] Faculty of Material Science and Engineering, Kunming University of Science and Technology, Kunming 650093, China

[7] Beijing Advanced Innovation Center for Materials Genome Engineering, University of Science and Technology Beijing, Beijing 100083, China

[8] Institute of Materials Intelligent Technology, Liaoning Academy of Materials, Shenyang 110167, China;

* Correspondence: jiabaorui@ustb.edu.cn (B.J.); shppmt@163.com (L.S.); qinml@mater.ustb.edu.cn (M.Q.)

† These authors contributed equally to this work.

Academic Editor: Antonino Gulino

Received: 8 December 2025

Revised: 27 December 2025

Accepted: 5 January 2026

Published: 12 January 2026

Copyright: © 2026 by the authors. Submitted for possible open access publication under the terms and conditions of the Creative Commons Attribution (CC BY) license.

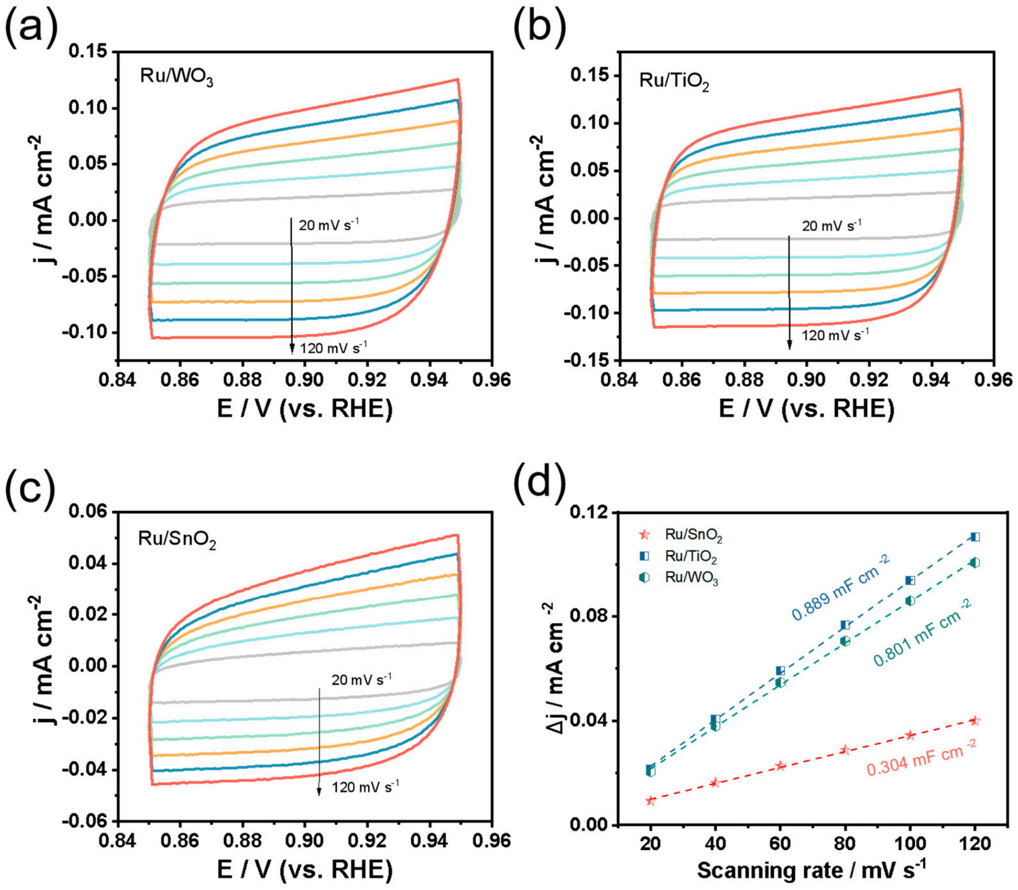

Figure S1. CV curves measured at different scan rates from 20 to 120 mV s^{-1} in 0.5 M H_2SO_4 for (a) Ru/WO_3, (b) Ru/TiO_2, (c) Ru/SnO_2 (d) Capacitive current at middle potential of CV curves as function of scan rates for Ru/WO_3, Ru/TiO_2 and Ru/SnO_2.

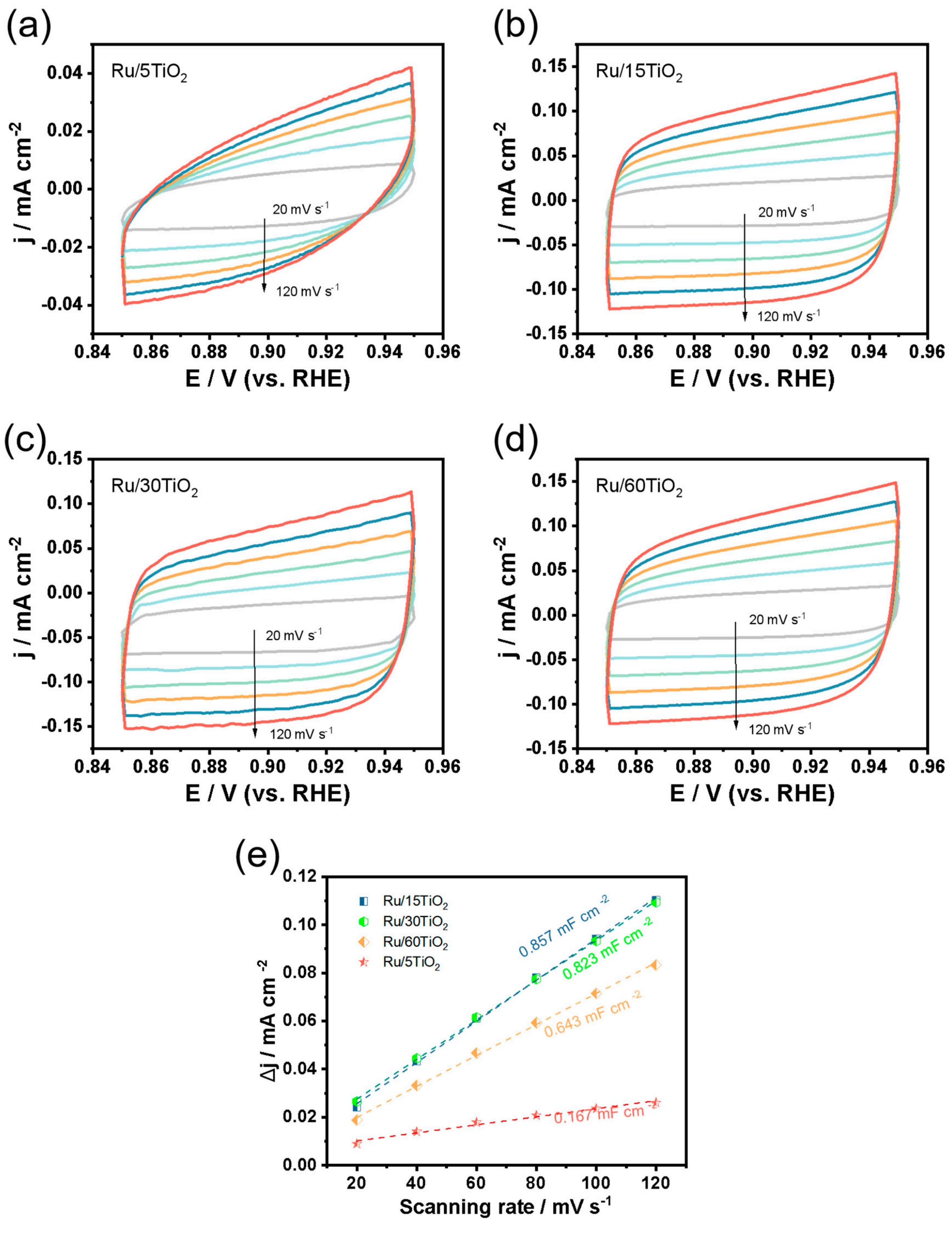

Figure S2. (a-d)CV curves measured at different scan rates from 20 to 120 mV s^{-1} in 0.5 M H_2SO_4 for different catalysts. (e) Capacitive current at middle potential of CV curves as function of scan rates for different catalysts.

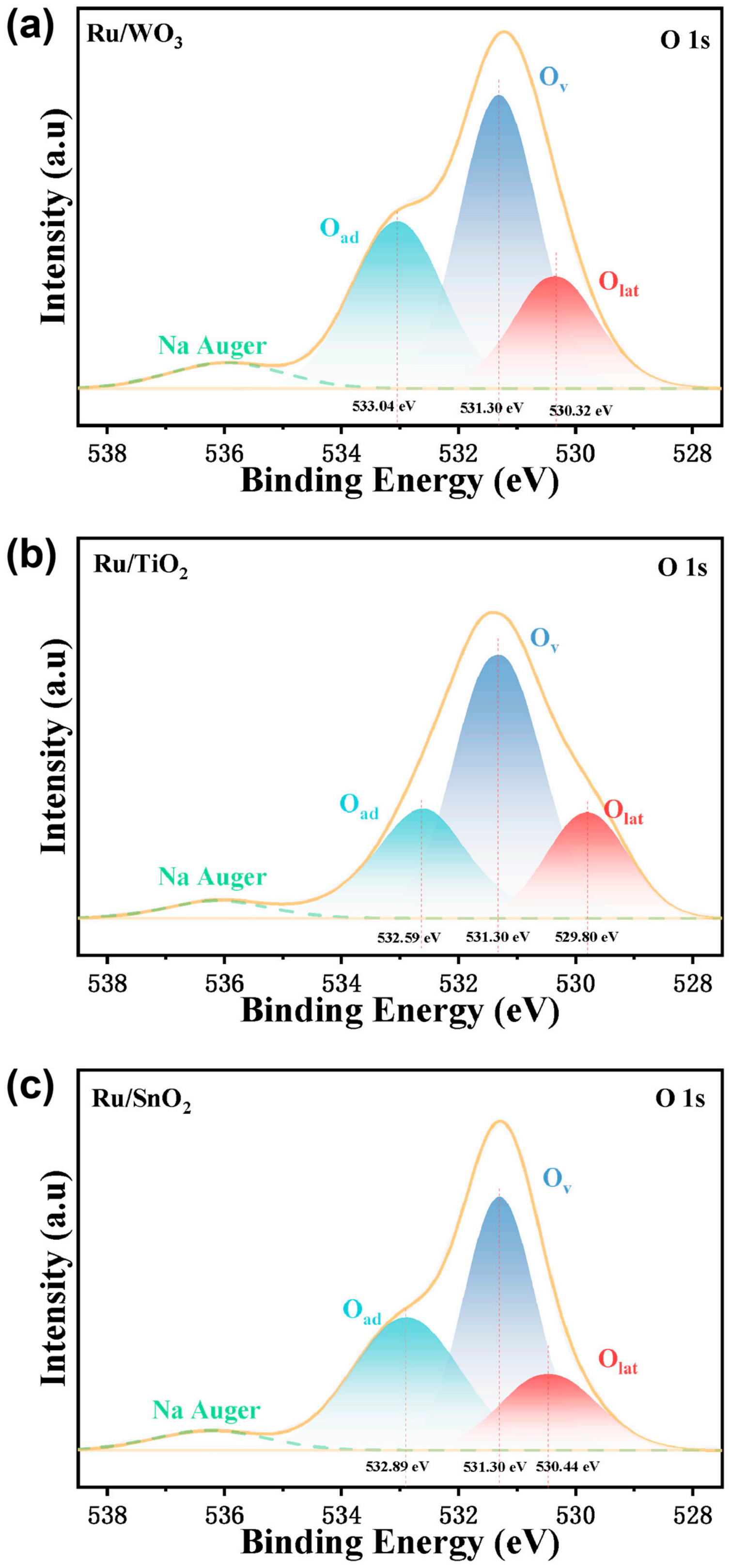

Figure S3. High-resolution O_{1s} XPS spectra of (a) Ru/WO_3, (b) Ru/TiO_2 and (c) Ru/SnO_2 catalysts.

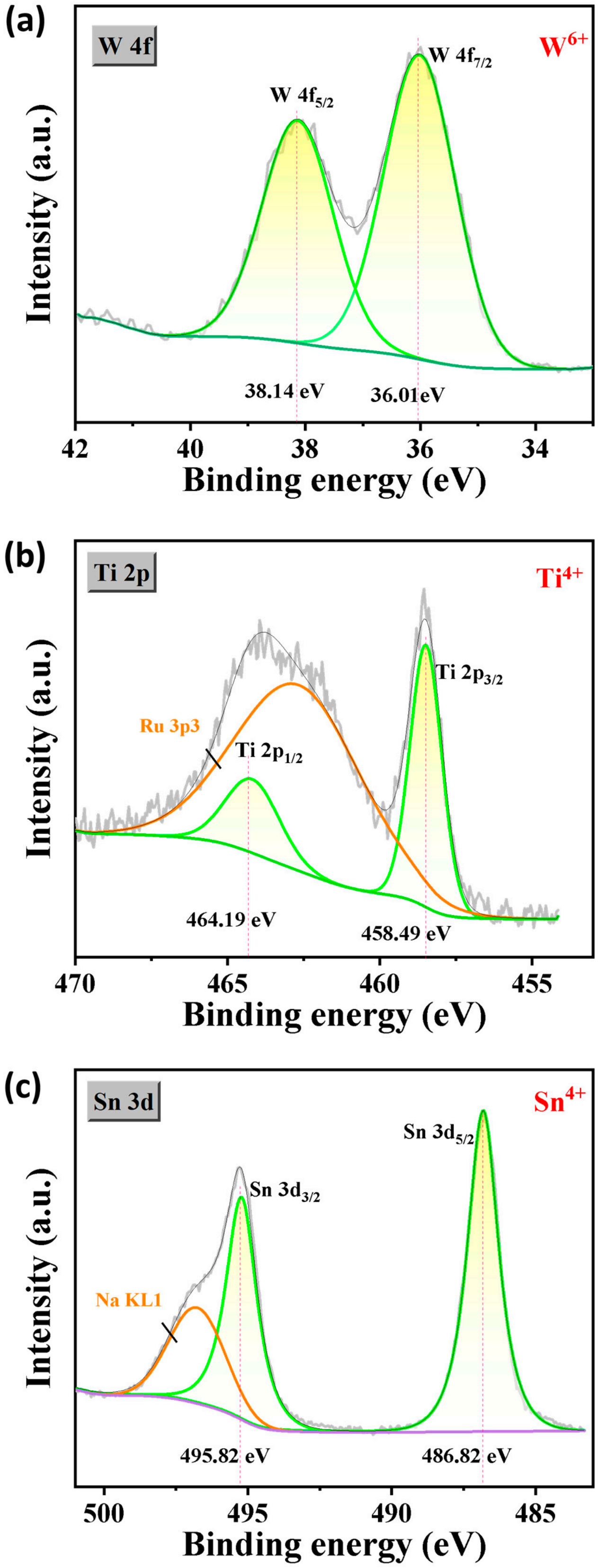

Figure S4. High-resolution XPS spectra of the support elements for Ru-supported oxide catalysts: (a) W 4f spectrum of Ru/WO_3,(b) Ti 2p spectrum of Ru/TiO_2, and (c) Sn 3d spectrum of Ru/SnO_2.

Table S1 ICP-MS analysis of the synthesized catalysts together with theoretical mass loadings. The theoretical values correspond to the wt% used in the synthesis.

Sample	Theoretical Ru [wt%]	ICP-MS Ru [wt%]
Ru/SnO_2	16.79	16.3642
Ru/TiO_2	16.79	16.5123
Ru/WO_3	16.79	16.1673

Table S2. Detailed Ru 3p peak-fitting parameters for the three catalysts.

Catalyst	Species	BE (eV)	FWHM (eV)	Area (CPS·eV)	Assignment
Ru/TiO_2	Ru^0	461.68	3.39	8596.86	Metallic Ru
	Ru^{4+}	464.66	3.38	3791.41	RuO_2-like
Ru/SnO_2	Ru^0	461.57	3.7	6512.38	Metallic Ru
	Ru^{4+}	464.25	3.87	4786.69	RuO_2-like
Ru/WO_3	Ru^0	461.93	3.13	4555.22	Metallic Ru
	Ru^{4+}	464.32	3.88	5237.23	RuO_2-like

Table S3. Detailed O 1s peak-fitting parameters for Ru/MO_x catalysts.

Catalyst	Species	BE (eV)	FWHM (eV)	Area (CPS·eV)	Content (%)	Assignment
Ru/TiO_2	O_{at}	529.8	1.64	15099.9	20.2	
	O_{def}	531.3	1.76	40158.24	**53.76**	O_{at} Lattice oxygen
	O_{ad}	532.59	1.89	19441.04	26.04	
Ru/WO_3	O_{at}	530.32	1.72	17216.08	20.46	O_{def}
	O_{def}	531.3	1.57	40014.69	**47.58**	Oxygen-vacancy-related oxygen
	O_{ad}	533.04	1.84	26864.13	31.96	
Ru/SnO_2	O_{at}	530.44	1.96	12952.64	18.52	O_{ad}
	O_{def}	531.3	1.45	31781.75	**45.45**	Adsorbed oxygen /–OH
	O_{ad}	532.89	2.18	25186.58	36.04	

Table S4 Binding energies of support element core levels for Ru-supported oxide catalysts.

**Catalyst**	**Core level**	**BE (eV)**	**Assignment**
Ru/TiO_2	Ti $2p_3/_2$	458.49	Ti^{4+}
	Ti $2p_1/_2$	464.19	
Ru/SnO_2	Sn $3d_5/_2$	486.82	Sn^{4+}
	Sn $3d_3/_2$	495.82	
Ru/WO_3	W $4f_7/_2$	36.01	W^{6+}
	W $4f_5/_2$	38.14	

Table S5 ICP-MS analysis of the synthesized catalysts together with theoretical mass loadings. The theoretical values correspond to the wt% used in the synthesis.

Sample	Theoretical Ru [wt%]	ICP-MS Ru [wt%]
Ru/5TiO_2	24.86	24.9705
Ru/15TiO_2	16.79	16.4978
Ru/30TiO_2	11.29	11.1842
Ru/60TiO_2	6.82	6.0484
